# Divergent Mutational Landscapes of Consensus and Minority Genotypes of West Nile Virus Demonstrate Host and Gene-Specific Evolutionary Pressures

**DOI:** 10.3390/genes11111299

**Published:** 2020-10-30

**Authors:** Haley S. Caldwell, Erica Lasek-Nesselquist, Paisley Follano, Laura D. Kramer, Alexander T. Ciota

**Affiliations:** 1 Department of Biomedical Sciences, State University of New York at Albany School of Public Health, Rensselaer, NY 12144, USA; haley.caldwell@health.ny.gov (H.S.C.); laura.kramer@health.ny.gov (L.D.K.); 2 The Arbovirus Laboratory, Wadsworth Center, New York State Department of Health, Slingerlands, NY 12159, USA; Paisley.Follano@ocfs.ny.gov; 3 Division of Genetics, Wadsworth Center, New York State Department of Health, Albany, NY 12208, USA; Erica.Lasek-Nesselquist@health.ny.gov

**Keywords:** viral evolution, arbovirus, flavivirus, West Nile virus, intrahost, interhost, virus adaptation

## Abstract

Our current understanding of the natural evolution of RNA viruses comes largely from consensus level genetic analyses which ignore the diverse mutant swarms that comprise within-host viral populations. The breadth and composition of viral mutant swarms impact viral fitness and adaptation, and the capacity for swarm plasticity is likely to be particularly important for arthropod-borne viruses (arboviruses) that cycle between taxonomically divergent hosts. Despite this, characterization of the relationship between the selective pressures and genetic signatures of the mutant swarm and consensus sequences is lacking. To clarify this, we analyzed previously generated whole genome, deep-sequencing data from 548 West Nile virus samples isolated from avian tissues or mosquitoes in New York State from 1999–2018. Both consensus level (interhost) and minority level (intrahost) nucleotide and amino acid sequences were analyzed, and diversity at each position was calculated across the genome in order to assess the relationship between minority and consensus sequences for individual genes and hosts. Our results indicate that consensus sequences are an inept representation of the overall genetic diversity. Unique host and gene-specific signatures and selective pressures were identified. These data demonstrate that an accurate and comprehensive understanding of arbovirus evolution and adaptation within and between hosts requires consideration of minority genotypes.

## 1. Introduction

The genus *Flavivirus* consists of arthropod-borne viruses (arboviruses) of significant public health importance, collectively causing over fifty million cases annually worldwide [1]. West Nile virus (WNV) is a mosquito-borne positive-sense single-stranded RNA virus of the family Flaviviridae and the genus *Flavivirus* [2]. It is considered an emerging infectious disease and is the most geographically widespread of the flaviviruses which include Zika (ZIKV), yellow fever (YFV) and dengue (DENV) viruses, among others [2,3]. Due to its rapid geographic spread and resultant public health burden in newly affected countries, WNV is considered to be among the most widespread and prevalent causative agent of viral encephalitis worldwide [4]. There are two primary lineages and up to five additional proposed lineages differing by as much as 20–25 percent nucleotide divergence. All Western Hemisphere strains belong to lineage 1a [3]. The ~11 kb genome shared among flaviviruses contains a single open reading frame encoding for three structural (C, prM, and E) and seven nonstructural proteins (NS1, NS2A, NS2B, NS3, NS4A, NS4B, and NS5) [5,6,7]. The NS3 and NS5 proteins are critical for WNV replication and consist of two domains each. The NS5 contains a methyltransferase (MTase) and an RNA-dependent RNA polymerase (RdRp) while the NS3 is composed of a helicase and a protease domain. Briefly, the RdRp of NS5 serves to replicate the WNV genome and the MTase provides a type 1 cap to the resultant positive sense genomes. The helicase of NS3 serves to unwind double stranded intermediates to allow asymmetric amplification of the positive strand [5,8,9,10].

The wide geographic range of WNV can in part be ascribed to the wide variety of susceptible hosts and competent vectors. WNV was introduced into the US in 1999, after which it became endemic. The public health burden has been substantial, with over 50,000 cases diagnosed, including 24,657 reports of neurologic disease and 2330 mortalities as of 2018, with an additional ~2000 cases reported per year [11]. Due to the high percentage of asymptomatic cases (~80%), estimates of the true caseload since 1999 is over three million [12,13]. WNV spread within the US can be attributed to the numerous highly competent *Culex* mosquitoes and over 300 susceptible bird species [3,14]. WNV is maintained in an enzootic cycle between these two hosts, where competent mosquitoes transmit the virus to avian hosts. Mammals are also capable of becoming infected with WNV but are generally considered dead-end hosts as viremia is insufficient for transmission to mosquitoes [2,15,16].

What facilities the rapid adaptation of WNV and other arboviruses to these vastly different host environments is not completely understood. One explanation highlights the error-prone nature of the RNA virus replication complex, which lacks proofreading activity and results in approximately 1 mutation per genome copied (10^−4^ mutations per nucleotide copied/~11 kb genome) [17]. As replication proceeds, more mutations accrue, resulting in a diverse swarm of closely related viral variants, which can confer unique fitness landscapes within and between divergent hosts [17,18]. Previous studies with WNV, St. Louis encephalitis virus (SLEV), chikungunya virus (CHIKV), human immunodeficiency virus, DENV, ZIKV, Japanese encephalitis virus, Venezuelan eastern encephalitis virus (VEEV), hepatitis C virus, and poliovirus (PV) have indicated that the magnitude and composition of variants within the mutant swarm alters viral fitness, virulence, immune evasion and tissue tropism in a host-dependent manner [19,20,21,22,23,24,25,26,27,28,29,30,31,32,33]. Studies altering the fidelity of the replicase within these viruses have highlighted the importance of the diversity of the mutant swarm in mediating viral virulence and fitness. Experimentally derived high and low fidelity mutants of WNV have differential fitness in avian and mosquito hosts. A high-fidelity mutant was shown to be less infectious in mosquitoes, but no difference in growth kinetics in avian cells was identified, suggesting that mutant swarm diversity is important in establishing infection in invertebrate hosts [23]. These results were mirrored with mutagen resistant SLEV, which was attenuated in mosquito but not vertebrate cell lines, presumably due to decreased mutant swarm diversity impeding escape from the invertebrate RNA interference (RNAi) immune pathway [22]. High fidelity PV mutants have been shown to have reduced neurotropism in mouse models, while high-fidelity CHIKV variants have decreased infectivity and dissemination in mosquitoes [34,35]. Low-fidelity VEEV variants exhibit increased immunogenicity and partial attenuation in mouse models, further solidifying the importance of diversity in host-specific fitness of arboviruses [24].

Despite the progress that has been made in understanding how mutant swarm diversity impacts aspects of viral fitness in experimental settings, our understanding of evolutionary history and genetic correlates of viral fitness in naturally occurring strains largely comes from studies which exclusively investigate consensus level genetic variability [36,37]. In addition, most publicly available whole-genome sequences contain only consensus data. However, multiple studies have called for increased scrutiny of minority variants for understanding how viral evolution affects persistence in diverse hosts and ecosystems, viral transmission, and antiviral escape [38,39,40,41]. Specifically, what remains unclear is if the benefits of diversity are host or gene-specific and if the selective pressures on minority genotypes are reflective of the variability identified in consensus sequence data. We utilized whole-genome, deep-sequencing data of WNV previously generated in our laboratory from isolates submitted to New York State (NYS) Department of Health for analysis from 1999–2018, to determine the relationship between minority and consensus genetic signatures. We hypothesized that consensus level variability would be a poor representation of mutant swarm breadth and that such comparisons would reveal biologically relevant selective pressures. Indeed, intrahost diversity was significantly increased compared to interhost diversity, and interhost diversity alone failed to indicate mutation hotspots which are likely to significantly impact viral fitness and alter host-specific viral adaptation and evolution.

## 2. Materials and Methods

To address how inter and intrahost diversity are related in WNV, we analyzed 548 whole-genome sequences with sufficient depth, out of a total of 588 possible isolates (Supplementary Table S1). Of these isolates, 543 were taken from surveillance samples of both WNV positive avian and equine tissues and whole mosquitoes submitted to the NYS Arbovirus Laboratory (Wadsworth Center, NYSDOH, NY, USA) from 1999–2015. The sequencing and processing of these samples were performed as in [42]. Sequences can be obtained via GenBank (BioProject #PRJNA262930). An additional 45 samples were obtained in a similar manner from 2013-2018 consisting of whole mosquitoes (GenBank Accession# MT967988- MT968032). Sequencing was performed as previously described in [42] using the Illumina MiSeq instrument (2 × 250 bp PE, Illumina, San Diego, CA, USA). 

Raw sequence reads from all 548 sequences were analyzed using Geneious Prime 2019.0.4. Bioinformatic analysis was performed using built-in Geneious applications unless otherwise indicated. Illumina reads were paired and merged (using BBmerge version 38.37 by Brian Bushnell) prior to trimming. Trimming was performed using the “Trim Ends” program with the error probability limit set to 0.05 and a minimum quality score of Q20. Ion Torrent PGM reads were trimmed using the same parameters. All samples were then mapped using the Geneious mapper on medium sensitivity and iterated up to five times to reference genome DQ164190, a lineage 1a WNV 02 isolates from New York State (GenBank: DQ164190.1). Variant calling was performed using “Find Variations/SNPs” under the following conditions: minimum coverage for a mutation to be called was 50x, and the minimum variant frequency was 2% or higher to account for sequencing errors inherent in both Ion Torrent PGM and Illumina MiSeq systems. *P*-values representing the probability of sequencing errors were calculated as part of the “Find Variations/SNPs” algorithm for each variant, and any variants with a *p*-value exceeding 10^−6^ were removed from the analysis. Variants were called relative to the reference sequence. Samples were run twice through an aggregation program, the first time with amino acid translation included to determine nonsynonymous mutations and then including single nucleotide variant calling to determine synonymous mutations. The resulting files, listing both amino acid and SNPs, were then aggregated using an in-house program, such that all consensus changes (mutations occurring in over 50% of reads at a given position) were contained within a single file and all minority changes (less than 50%) in a separate file for all 548 sequences. An additional in-house program was developed to perform the same operation on the amino acid changes only. This analysis was performed on all sequences combined, sequences originating from avian tissues (276) and sequences originating from mosquitoes (270). As an additional check for data quality, the diversity and depth of all isolates were compared via Spearman’s Rank Coefficient to determine whether sequence diversity was related to depth and the results was not significant (r = 0.005, *p* = 0.6322) indicating that diversity was not related to depth at the ends of the PCR fragments used to amplify samples for sequencing.

The aggregated output files thus consisted of both inter (consensus) and intra (minority) host nucleotide (NT) and amino acid (AA) variations for combined samples, avian samples and mosquito samples. Shannon entropy (S_n_) was calculated for each coding position in the genome in each of the 12 aggregated files (all NT interhost, all NT intrahost, all AA interhost, all AA intrahost, avian NT interhost, avian NT intrahost, avian AA interhost, avian AA intrahost, mosquito NT interhost, mosquito NT intrahost, mosquito AA interhost, and mosquito AA intrahost) utilizing the following formula: S_n_ = sumPi(lnP_i_)/lnN, where P_i_ is the frequency of the variant at a given position and N is the total number of sample sequences. Substitution bias analysis was performed by determining the number of specific mutations within each of the 12 sample sets as a proportion to the total number of mutations (NT or AA substitutions) and compared using a Fischer’s exact test to predicted mutations (based on a transition to transversion ratio of 2:1 and assuming equal distribution of transitions and transversions). Inter and intrahost S_n_ at each position was compared for each host and for both NT and AA datasets using both a Spearman’s rank correlation and a simple linear regression. All statistical analysis with the exception of S_n_ was performed in Graphpad Prism 8.0.2. 

## 3. Results

### 3.1. Host and Gene-Specific Genetic Diversity and Selective Pressures

Genome-wide analysis of 270 mosquito isolates and 276 avian isolates of WNV identified a total of 25,503 unique single nucleotide polymorphisms (SNPs) and 4702 unique AA substitutions on the interhost level, and a total of 380,537 unique SNPs and 151,265 unique AA substitutions on the intrahost level. As expected, diversity was significantly higher on the intrahost level. Overall S_n_ values for NT and AA were 2.00 × 10^−3^ and 5.00 × 10^−4^ respectively, for interhost, and 3.46 × 10^−2^ and 1.92 × 10^−2^, respectively, for intrahost. Proportion of mutations which were nonsynonymous (pN) were 1.77 × 10^−1^ and 1.91 × 10^−1^ for avian and mosquito datasets, respectively, consistent with the dominance of purifying selection. Interhost S_n_ was statistically equivalent for avian and mosquito isolates on both NT and AA levels. Intrahost S_n_, on the other hand, was significantly higher for avian isolates relative to mosquito isolates (Table 1; Fisher’s exact test, *p* < 0.001). 

**Figure 1:**
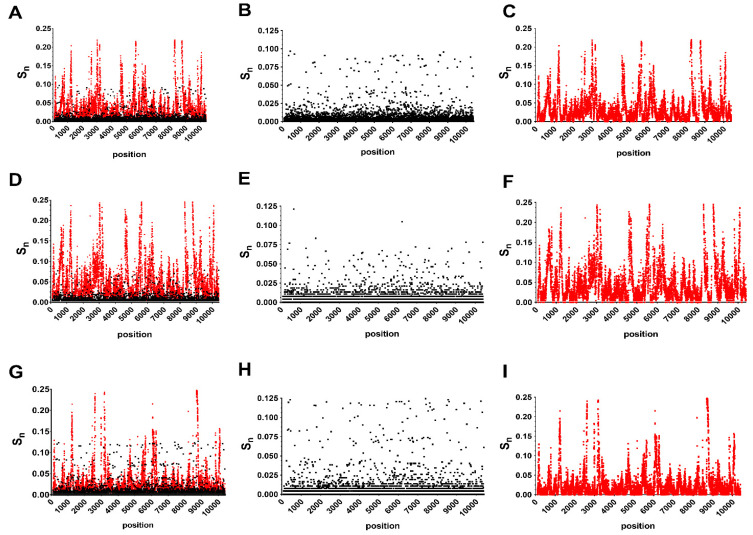
Unique mutational landscapes of West Nile virus in consensus and minority sequences. Comparison of inter and intrahost nucleotide (NT) entropy (S_n_) at each position in the genome for all hosts (**A**–**C**), avian hosts (**D**–**F**) and mosquito hosts (**G**–**I**). Panels B, E, and H show interhost diversity while panels C, F, and I show intrahost diversity.

**Figure 2:**
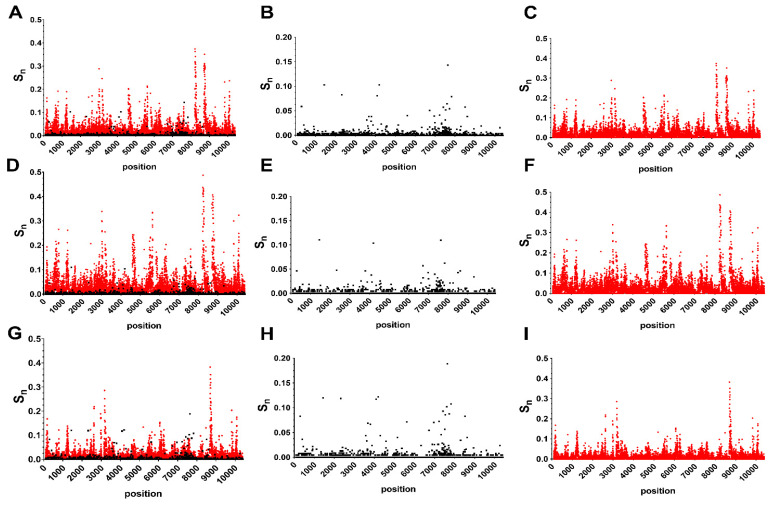
Unique amino acid (AA) landscapes of West Nile virus identified in consensus and minority sequences. AA entropy (Sn) in all hosts (**A**–**C**), avian hosts (**D**–**F**) and mosquito hosts (**G**–**I**) is shown at each position in the genome. Interhost AA diversity is shown in panels B, E, and H while intrahost diversity is shown in panels C, F, and I.

Position-specific S_n_ calculations revealed diversity hotspots dispersed throughout the genome in both consensus and minority genotypes (Figure 1). Individual positions with high levels of variability are more distinct on the amino acid level (Figure 2). A total of 13 AA positions were found to have S_n_ levels above 0.05 in combined data (Figure 2D).

**Figure 3:**
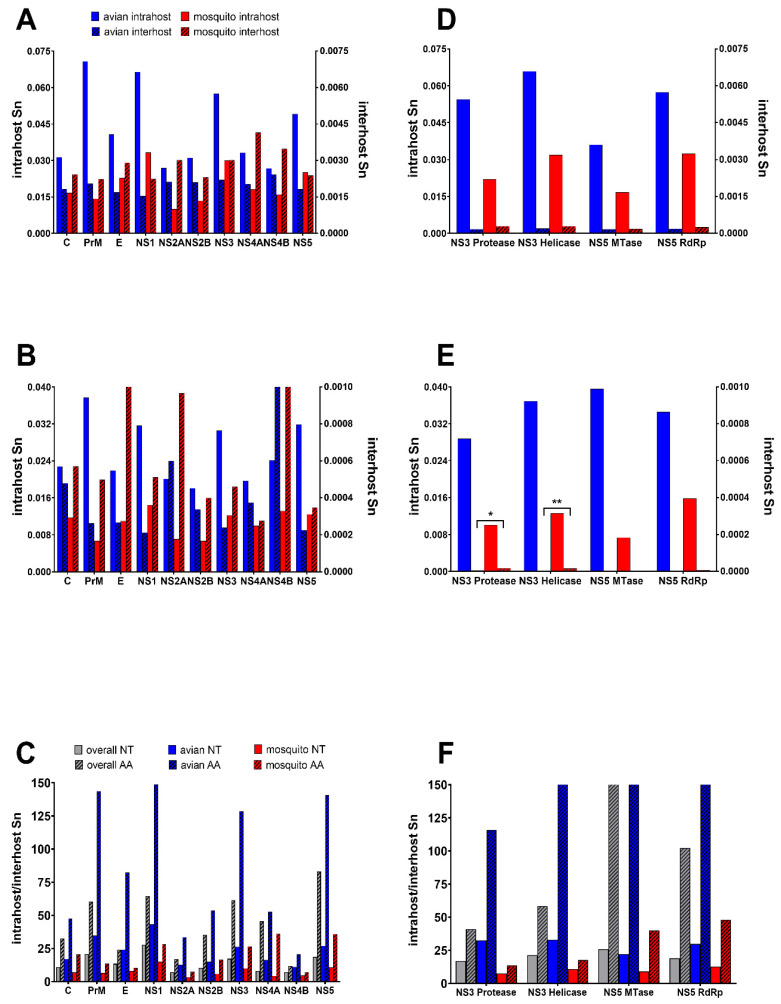
Gene-specific and host-specific differences for West Nile virus (WNV) inter and intrahost genetic diversity. Mean entropy (Sn) per gene for inter and intrahost nucleotides (NT) (**A**), and amino acids (AA) (**B**), is shown in the context of subunits of the NS3 and NS5 genes for NT (**D**) and AA (**E**). Interhost Sn was compared between avian and mosquito hosts for each gene and subunit using a Welch’s T-test where ** *p* < 0.005 and * *p* < 0.05. Intrahost S_n_ was also compared; however, all relationships were significant (*p* < 0.0001). The ratio of intra to interhost NT data for each gene (**C**) and each subunit within NS3 and NS5 (**F**).

For avian-only genotypes, there were a total of 5 positions with S_n_ levels above 0.05, including 1442, 4195, 6721, 7635 and 7826 (Figure 2E). These same 5 positions and an additional 14 were identified in mosquito genotypes (295, 2321, 3680, 3808, 4088, 5611, 6721, 6956, 7226, 7419, 7515, 7527, 7602, 7731, and 8511; Figure 2F). To further unravel host-specific differences and identify areas with high diversity, gene-specific AA and NT S_n_ were calculated and compared. Two genes with high diversity in both hosts (NS3 and NS5) were further divided into subunits (Figure 3). Interhost NT diversity was relatively similar among genes, yet the highest levels were identified in the NS4 genes (Figure 3A). In the case of NS4B, this translated to high levels of AA diversity for both avian and mosquito isolates (Figure 3B). Relatively high levels of AA interhost diversity were also measured for both hosts in the C and NS2A genes. Interhost AA diversity of the E gene, on the other hand, was host-specific, with significant diversity measured in mosquito isolates and relative conservation in avian isolates. Host-specific differences were much more apparent with intrahost data. Avian-specific intrahost diversity for both AA and NT levels were highest in PrM, NS1, NS3 and NS5, while mosquito-specific intrahost diversity was highest in NS1 and NS4B (Figure 3A). The highest areas of AA specific intrahost diversity in mosquitoes include E and NS2A while avian isolates showed increased AA intrahost diversity at NS4A (Figure 3B). In terms of NS3 and NS5, increased mosquito intrahost diversity occurred in the NS3 helicase and NS5 RdRp while increased avian intrahost diversity was measured for each subunit in NS3 and NS5 with both NT and AA data (Figure 3D,E). For the interhost comparison, significant differences between mosquito and avian AA diversity in the NS3 protease and NS3 helicase were measured (two-way ANOVA with Sidak’s multiple comparisons test, *p* < 0.05). For the intrahost comparisons, all tests comparing avian and mosquito gene and protein diversity to one another demonstrated significant differences (two-way ANOVA with Sidak’s multiple comparisons test, *p* < 0.0001). 

### 3.2. Unique Intra and Interhost Genetic Signatures

**Figure 4:**
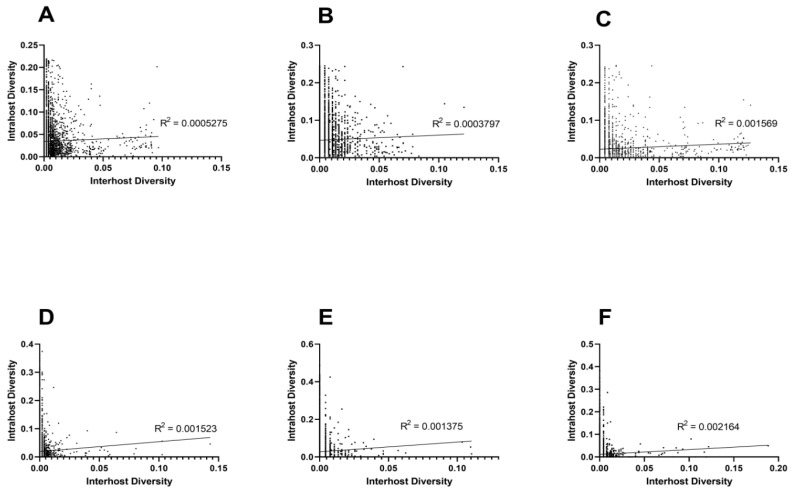
Weak correlation between inter and intrahost genetic diversity (S_n_) of WNV within all hosts (**A**,**D**), avian (**B**,**E**), and mosquito hosts (**C**,**F**) for NT S_n_ shown in panels A-C and AA S_n_ shown in panels D-F. Linear regression analysis was performed on each data set, and the resulting R^2^ is shown. A Spearman’s rank correlation was also performed in each case, indicating weak but statistically significant (*p* < 0.05) relationships for each data set.

The correlation between inter and intrahost genetic diversity was weak for individual host and combined data (Figure 4; Linear regression analysis, *p* < 0.01, R^2^ < 0.003; Spearman’s rank correlation, *p* < 0.005) for both NT (Figure 3A–C) and AA (Figure 4D–F). Together, these data clearly demonstrate that intrahost diversity does not reliably predict interhost diversity in either host. 

There were numerous individual positions or regions with high levels of NT intrahost diversity that were relatively conserved on the consensus level (Figure 1 and Figure 2). For example, the three most diverse individual positions, 8283-8285 (S_n_ = 0.22) were 100% conserved on the consensus level. Similar discrepancies exist throughout the genome, particularly at individual positions in the NS1, NS3 and NS5 genes (Figure 1). This included the most diverse regions of the NS5 consisting of ~100bp stretches beginning at positions 8230 and 8740. Interestingly, mutations in these regions are largely nonsynonymous and host-specific, with the region of diversity beginning at position 8230 being unique to avian isolates. To further clarify these differences, S_n_ ratios (intrahost/interhost) between hosts by gene and subunit were also compared (Figure 3C,F). Avian interhost S_n_ was significantly different than intrahost S_n_ at many genes, including PrM, E, NS1, NS3 and NS5, as well as in the NS3 and NS5 subunits (Figure 3C,F; two-way ANOVA with Sidak’s multiple comparisons test, *p* < 0.0001). Mosquito interhost S_n_ values more closely mirror intrahost S_n_ across the genome, with the exceptions of NS4A and NS5 (Figure 3C,F). The proportion of nonsynonymous mutations (pN) was significantly higher in intrahost data as compared to interhost (0.398 vs 0.184; Fisher’s exact test *p* < 0.0001). 

To determine how individual substitutions contributed to diversity at both inter and intrahost levels, the actual number of transitions and transversions was compared to the predicted number of transitions and transversions based on the total number of SNPs in avian and mosquito hosts. All tests were statistically significant, indicating a deviation from the expected transition to transversion ratio (2:1), and additional biases were identified in minority genotypes relative to consensus genotypes. For both avian and mosquito samples, interhost transitions were higher than expected, with a ratio of 6.7:1 and 6.9:1, respectively. Individual mutations were also compared in the same manner between hosts. However, due to the use of DQ164190 as a reference strain, not all resulting mutation were necessarily single step and may exclude previous mutations and/or reversions. Again, nearly all comparisons were highly significant (*p* < 0.0001) with the exception of mosquito interhost A-U mutation (*p* > 0.05) and avian interhost U-A mutation (*p* < 0.05), indicating that transition and transversion mutations are not uniformly distributed across the samples (Table 2). Host-specific differences did exist between mosquito and avian mutations, with all avian interhost transitions being higher than expected, while only certain interhost mosquito transitions being higher than expected (U-C and C-U) while others were lower than expected (A-G and G-A). Direct comparison of avian and mosquito substitution bias was also highly discrepant between the interhost and the intrahost data (Table 3). There were only two mutations (C-U, C-G) where the interhost results agreed with the intrahost while the remaining differed in either host bias (avian or mosquito) or significance (Table 3). It is noteworthy that the inter and intrahost mutations in avian hosts show precisely opposite relationships between transitions and transversions, with interhost having more transitions and intrahost more transversions.

## 4. Discussion

Arboviruses like WNV can adapt readily to highly disparate hosts despite differing selective pressures and bottlenecks imposed during host switching [43]. Minority variants comprising the mutant swarm of RNA viruses are thought to play an important role in this rapid and diverse adaptability [40,44,45]. However, analysis of minority variants in flaviviruses and other RNA viruses have generally been lacking despite their pivotal role in fitness, virulence, and transmission. Here, we present a deep-sequencing analysis of 548 WNV isolates from both avian and mosquito hosts comparing consensus (interhost) and minority (intrahost) genotypes at both the nucleotide and amino acid level. Our results show that inter and intrahost genetic signatures are highly distinct. 

Genetic diversity across the WNV genome was highly host-specific. While consensus level diversity was higher among mosquito isolates, this difference was not statistically significant. However, we would not expect host bias on the interhost level, because consensus level differences do not fluctuate significantly between matched hosts. Intrahost diversity, on the other hand, was significantly higher in avian isolates at both NT and AA levels. Decreased interhost avian diversity is in agreement with NT diversity as in [46] and [36] but in contrast to several smaller studies which utilized either experimentally derived isolates [47], fewer isolates, or only determined intrahost diversity for a smaller portion of the genome [37,48,49,50,51]. Our results are unlikely to be due to differences in read-depth caused by primer-based PCR amplification of genomic fragments [52]. In addition, while process errors (from amplification and sequencing) are an important consideration (particularly for low-frequency mutations), all samples had similar viral titers and were subjected to identical methodology [42]. Lastly, SNPs with frequencies under 2.0% were excluded from the analyses. The majority of higher diversity regions were not identified in primer bindings sites, and overall diversity in these regions did not exceed surrounding regions. Although positions identified as high diversity including 8230 to 8740 and 8511 are located within primer sites, overlapping amplicons utilizing unique primer pairs identified similar levels of diversity in these regions, increasing confidence that these represent accurate measures of genome variation originating in virus isolates [42]. However, despite isolates being taken from the same location over time, many of which are matched by year, differences in diversity independent of host origin are certainly plausible especially considering that many of the mosquito samples were taken after 2010 while the majority of bird samples are from prior years.

Across the entire genome and regardless of host origin, interhost diversity was poorly predictive of intrahost diversity. In fact, some of the most diverse intrahost regions were perfectly conserved in the interhost data. These areas of high intrahost diversity are critical in understanding viral evolution, as minority variants have been shown to interact cooperatively, altering viral fitness and pathogenicity [35,53]. Further, altered mutant swarm diversity is implicated in tissue tropism, neuropathogenicity and host-specific adaptation [22,23,35]. Characterization of the mutant swarm is thus paramount to understanding the potential mechanisms of host-specific adaptation and predicting phenotypic variation of circulating or newly emerging flaviviruses. 

Perhaps most significantly, inter and intrahost diversity differed in individual genes and subunits. While previous studies have made broad conclusions regarding host-specific selective pressures and diversity, genetic correlates of viral fitness, and adaptive or evolutionary trade-offs, the extent to which these characteristics are region-specific has rarely been considered [15,54]. Regions with significant mutant swarm breadth that are relatively conserved on the consensus level are likely subjected to either density-dependent selection or distinct, host-specific pressures. Sites of high WNV intrahost variation specific to each host include PrM, NS1, NS3, and NS5 for avian isolates and NS3 and NS5 for mosquito isolates. High interhost variation in mosquito samples was present in the E and NS2A, although this variation is unlikely to be due to host-specific selective pressures at these sites. While characterizing the mechanistic basis for these differences requires additional studies, some insights can be made. Interestingly, the areas of high NT diversity did not always correlate with areas of high AA diversity, suggesting gene-specific differences in the strength of purifying selection. Areas of high AA diversity include E, NS2A and NS4. The intrahost exclusive host-specific diversity hotspots include regions in the PrM, NS1, NS2A, NS3 and NS5, which have been shown to be significant in viral fitness. The envelope protein is critical for infection of targeted cells and thus viral replication and is explicitly implicated in host range and tissue tropism, as well as acting as the primary antigen for inducing immunity [55,56]. The PrM and E protein mediate replication in avian hosts, and substitutions in these proteins can result in attenuation of WNV in American crows, house sparrows and *in vitro* [57]. NS1, which is a known virulence factor when secreted and is involved in viral replication and immune evasion in mammalian hosts [58,59,60], could conceivably be involved in avian immune modulation. It has also been previously shown to have high levels of intrahost diversity [52]. The E, prM, and NS1 proteins are all antigenic in the host [56,61,62,63]. It is plausible, therefore, that there is selection for amino acid diversity to evade the adaptive immune response. Understanding the extent of this variation has critical importance for the design of antiviral strategies that rely on antibody binding. 

Both NS4A and NS2A have high intrahost AA diversity and are known to inhibit IFN signaling acting to suppress the host immune system [64,65]. NS2A has been specifically implicated in dengue for suppression RNAi responses in mammalian and mosquito cells [66]. Therefore, the high intrahost AA diversity in mosquito isolates could be in response to selection pressure via the mosquito RNAi response, allowing for increased immune escape in these hosts. NS4A is an essential component of the replicase and has been found to induce autophagy in epithelial cell to ensure survival, however its role in host-specific fitness is not known and requires further study [67,68]. Previous studies have shown that point mutations within NS4A can differentially regulate autophagy [69]. Thus, increased avian AA diversity in NS4A could have multiple benefits including increased suppression of the host immune system or host-specific modulation of autophagy to optimize fitness. Previous studies have shown positions in the NS2A, NS4A and NS5 to be under positive selection, further supporting their importance in viral fitness [15,42,70]. Additionally, altered diversity within specific genes between mosquito and avian hosts could represent host-specific differences in the strength of purifying selection, the strength of density-dependent selection or the frequency and size of bottlenecks. For example, increased diversity within E and NS2A proteins of mosquito isolates may be due to relaxed purifying selection resulting from the lack of an adaptative immune system in mosquitoes [55,56,64,65].

NS3 and NS5 are the primary drivers of flaviviral replication and mutations in NS5 can alter fidelity and thus, mutant swarm diversity [19,23,35,71]. Although both genes are typically highly conserved due to their function, differences in diversity between hosts are not necessarily surprising considering their pivotal role in generating diversity [42,72]. Previous studies have also demonstrated that altered polymerase function and fidelity may be differentially selected in disparate hosts [23,73]. The implication of different subunits within NS3 and NS5 in host-specific fitness is intriguing, as it could reveal unique host-specific selective pressures during viral replication. Further, previously unrecognized sites of diversity within specific hosts including NS4A may reveal novel functions of viral proteins. 

Comparison of mutational frequencies of mosquito and avian isolates revealed additional differences in substitution bias between consensus and minority genomes. Specifically, intrahost analysis revealed a higher ratio of transversions and nonsynonymous mutations relative to consensus genotypes. Our results thus support the selective hypothesis for explaining the transition-transversion substitutional bias. Simply, the selective hypothesis states that transitions are more likely to result in conservative amino acid changes, which are more likely to maintain viral fitness [74,75]. Fixed mutations would therefore be more likely to be transitions due to the conservation of function. Minority mutations, however, could have more flexibility in disrupting protein function as well as a higher tolerance for nonsynonymous mutations, which could be particularly beneficial for arboviruses cycling through taxonomically distinct hosts. Cooperative interactions within the mutant swarm could rescue the biochemical function of these minority variants in such a case. This notion is supported by Lyons and Lauring, 2017, which noted significant detrimental effects of transversions in influenza virus fitness [76]. This idea may also explain the disparities between individual host biased mutations in the interhost and intrahost analysis. Differential codon usage could also contribute to host-biased mutations, yet a study by Moratoria et al., 2013 reported that relative synonymous codon usage was similar in 449 WNV strains isolated from birds, equines, humans and mosquitoes [77]. 

## 5. Conclusions

Our results demonstrate the importance of analysis of both intra and interhost level sequencing data for arboviruses. Consensus sequences are clearly poor representations of mutational landscapes, and therefore do not accurately reflect diversity hotspots, substitution bias, or host-specific selective pressures. A large-scale study by Parameswaran et al. analyzed whole-genome intrahost diversity for DENV highlighted differences between inter and intrahost genotypes, although it focused exclusively on isolates from human sera [78]. In agreement with our analysis, intrahost analysis revealed gene-specific variation in diversity and substitution bias between hosts that were not present in consensus sequences. Together these data clearly demonstrate that accurate characterization of arbovirus adaptation and evolution requires tools and analyses that consider the full extent of genotypes that comprise these viruses within and between hosts.

## Figures and Tables

**Table 1:** Whole-genome average intra and interhost entropy (S_n_) for each host.

**Interhost**					
	**AA ^1^**	**NT ^2^**	**pN ^3^**	**Mean S_n_ NT**	**Mean S_n_ AA**
All	4702	25,503	0.1844	0.0023	0.0005
Avian	1896	10,739	0.1766	0.0019	0.0004
Mosquito	2796	14,671	0.1906	0.0028	0.0007
**Intrahost**					
	**AA ^1^**	**NT ^2^**	**pN ^3^**	**Mean S_n_ NT**	**Mean S_n_ AA**
All	151,265	380,537	0.3975	0.0346	0.0192
Avian	113,728	283,958	0.4005	0.0470 *	0.0277 *
Mosquito	36,449	91,853	0.3968	0.0233 *	0.0114 *

^1^ indicates number amino acid substitutions, ^2^ indicates the number of nucleotide substitutions, and ^3^ indicates the proportion of nonsynonymous mutations. * indicate statistically significant differences (*p* < 0.0001) between intrahost avian and mosquito mean nucleotide (NT) and AA (amino acid) diversity via Welch’s unpaired T-test with Tukey’s post-correction.

**Table 2:** The proportion of each type of substitution within inter and intrahost datasets for each host.

	**Intrahost Substitution Ratio**											
	**# A - U**	**# A - C**	**# A - G**	**# U - A**	**# U - G**	**# U - C**	**# G - A**	**# G - C**	**# G - U**	**# C - U**	**# C - G**	**# C - A**
All	0.0966	0.0866	0.0954	0.0674	0.0597	0.0777	0.0948	0.0910	0.0934	0.0932	0.0717	0.0726
Avian	0.0969	0.0868	0.0943	0.0671	0.0597	0.0770	0.0943	0.0935	0.0943	0.0918	0.0715	0.0727
Mosquito	0.0957	0.0858	0.0995	0.0679	0.0594	0.0799	0.0967	0.0834	0.0903	0.0971	0.0724	0.0718
	**Interhost Substitution Ratio**											
	**# A - U**	**# A - C**	**# A - G**	**# U - A**	**# U - G**	**# U - C**	**# G - A**	**# G - C**	**# G - U**	**# C - U**	**# C - G**	**# C - A**
All	0.0462	0.0151	0.1087	0.0325	0.0038	0.2592	0.1169	0.0031	0.0151	0.3879	0.0013	0.0100
Avian	0.0549	0.0092	0.1187	0.0359 ^ns^	0.0049	0.2701	0.1104	0.0023	0.0131	0.3716	0.0009	0.0076
Mosquito	0.0398 *	0.0195	0.1018	0.0300	0.0030	0.2508	0.1219	0.0037	0.0167	0.3998	0.0016	0.0116

All avian and mosquito mutations were compared to expected outcomes via Fisher’s exact Test. ^ns^ refers to nonsignificant and * *p* < 0.05. All other comparisons between avian and mosquito mutations and expected outcomes were highly significant (*p* < 0.0001).

**Table 3:** Host bias of individual nucleotide substitutions.

Interhost		Intrahost	
Mutation	Significance	Mutation	Significance
# A - U	a ****	# A - U	^ns^
# A - C	m ****	# A - C	^ns^
# A - G	a ****	# A - G	m ****
# U - A	a **	# U - A	^ns^
# U - G	a *	# U - G	^ns^
# U - C	a ****	# U - C	m **
# G - A	a ****	# G - A	m *
# G - C	^ns^	# G - C	a ****
# G - U	m *	# G - U	a ***
# C - U	m ****	# C - U	m ****
# C - G	^ns^	# C - G	^ns^
# C - A	m **	# C - A	^ns^

All avian and mosquito mutations were compared to each other via Fisher’s Exact test, **** *p* < 0.0001, *** *p* < 0.001. ** *p* < 0.01, * *p* < 0.05 and ^ns^ is nonsignificant. Avian (a) or mosquito (m) indicates host bias for that mutation.

## Supplementary Materials

The following are available online at https://www.mdpi.com/2073-4425/11/11/1299/s1, Table S1: New York State Surveillance Isolates Analyzed.
