# Supplementary material for: Divergent Mutational Landscapes of Consensus and Minority Genotypes of West Nile Virus Demonstrate Host and Gene-Specific Evolutionary Pressures
Source: Genes (Basel). 2020 Oct 30;11(11):1299. doi: 10.3390/genes11111299 (PMC7692055; doi:10.3390/genes11111299)
Supplement: Supplementary file 1 [file genes-11-01299-s001.pdf]

Supplementary Table 1: New York State Surveillance Isolates Analyzed

Year	# Mosquito	# Avian	# Other	Total
**1999**	1	3	2	6
**2000**	5	2	0	7
**2001**	6	8	2	16
**2002**	28	35	3	66
**2003**	26	29	3	58
**2004**	9	49	0	58
**2005**	14	38	0	52
**2006**	15	34	0	49
**2007**	12	33	0	45
**2008**	13	25	0	38
**2009**	11	22	0	33
**2010**	13	7	0	20
**2011**	24	0	1	25
**2012**	32	2	0	34
**2013**	33	0	0	33
**2014**	9	0	0	9
**2015**	10	0	0	10
**2016**	6	0	0	6
**2017**	8	0	0	8
**2018**	15	0	0	15
			Total	588
